# Exploring the Use of Facet Joint Injections Through Instagram

**DOI:** 10.7759/cureus.38554

**Published:** 2023-05-04

**Authors:** Halil C Kose

**Affiliations:** 1 Pain Medicine, Kocaeli City Hospital, Kocaeli, TUR

**Keywords:** online platform, digital health awareness, social media platform, chronic pain management, content analysis, health communications, instagram, facet pain, internet, facet joint injection

## Abstract

Purpose: Facet joint injection (FJI) is a minimally invasive procedure used to relieve pain and inflammation in the facet joints of the spine. In light of the growing presence of social media, it is essential to comprehend its effects on the healthcare industry. Little is known about how FJI is discussed on Instagram. The purpose of this study was to investigate the characteristics and production sources of FJI-related posts on Instagram.

Methods and materials: This study provides a descriptive analysis of Instagram posts with the keywords #facetjointinjection, #facetjointinjections, #facetinjection, #facetinjections, #facetblock, and #facetblocks on March 1, 2023. The results were categorized by source into four groups; posts created by healthcare professionals (surgeon/non-surgeon), medical organizations, patients, or not otherwise specified. The content was categorized by type (educational and patient/physician experience) and user influence (number of followers and posts).

Results: The search resulted in 2718 posts. Most post uploaders were mainly physicians (43.1%, n = 275). The distribution of remaining Instagram users with FJIs posts was as follows: 27.1% (n=173) patients, 16.3% (n=104) medical organizations, and 13.4% (n=86) not otherwise specified. Among the posts, 1136 (41.7 %) were from accounts created by patients, 1015 (37.3%) by physicians, 441 (16.2%) by medical organizations, and 126 (4.6%) were unspecified. The analysis showed a significant difference in the distribution of posts among patients and physicians, as well as patients and other unspecified groups (p<0.05). The reported side effects were as follows: pain around the injection site (36%), swelling (17%), weight gain (15%), and anxiety (32%).

Conclusions: This study demonstrates that physicians are widely present on social media. However, when searching for posts about facet joint interventions, posts written by patients are more likely to be seen by the public. The results of this article emphasize the impact physicians have on online platforms and the need to raise FJI awareness on Instagram. Due to a lack of information and their anxiety about the unknown, patients have voiced hesitation about undergoing FJIs. To address this issue, it is the responsibility of physicians to enhance the accessibility of accurate information for patients in order to alleviate their anxiety. Additionally, reputable pain medicine societies and qualified specialists should upload credible posts on facet joint interventions that include accurate information, high-quality images and videos, and proper scientific commentary, with the aim of enhancing the quality of online health information.

## Introduction

Lumbar and cervical facet joints are a known cause of chronic axial spinal pain, which can result in significant disability and healthcare expenditures [1,2]. Facet joints that connect adjacent vertebrae always have a function in limiting spine movements, and their function in loadbearing becomes more pronounced as discs get older and degenerate [3]. Patients experiencing facet joint pain may manifest symptoms such as neck pain, back pain, and exacerbated discomfort during hyperextension, lateral bending, and rotation. The facet joint injection (FJI) has emerged as a beneficial technique in both diagnosing facet joint pain and providing therapeutic relief [4].

The Internet has revolutionized the world and resulted in innovative advances in the education and dissemination of medical information. It has become one of the most important sources of information for medical issues. Instagram is a popular image-based social media site with over 1 billion users [5]. Given the unique role of visuals in communication and the importance of framing messages, Instagram and other visual-based platforms could prove to be particularly effective in health communication and discourse monitoring [6]. Previous research has shown that messages incorporating visual elements are more likely to be retained, remembered for a longer period of time, and accurately recalled compared to those consisting solely of text [7]. Patients who post their medical experiences online create support networks and use social media to console and provide comfort to other patients with the same disease. With the aid of hashtags, patients can more easily access posts regarding their disease from other patients or medical professionals.

FJI comprises one of the most common procedures performed in interventional pain practices. As more patients experience back and/or neck pain, there is a growing interest in treatments such as FJI among patients. The aim of this manuscript is to evaluate the content on FJI provided on Instagram by patients, physicians, and medical organizations.

## Materials and methods

This study provides a descriptive analysis of Internet searches, without involving any experimental humans or animals. As a result, since the evaluated data are readily available to the public, this study does not need ethics committee approval.

On March 1, 2023, the hashtags #facetjointinjection, #facetjointinjections, #facetinjection, #facetinjections, #facetblock, and #facetblocks were searched using the Instagram tool. All information was accurate as of that day, and only posts in English were evaluated. Duplicate and inaccessible posts were eliminated. The posts were categorized into four groups: physicians (surgeon [orthopedic surgeon or neurosurgeon] and non-surgeon [interventional radiology, physical medicine and rehabilitation, anesthesiology, and pain physician]), patients, medical organizations, or not otherwise specified. The information was divided into categories based on its type (education or patient/physician experiences) and the influence of users (number of posts, number of followers). All captions and hashtags for patient postings were examined, and the statistical analysis of those posts was performed. The distribution of followers and messages between the users was analyzed.

SPSS Statistics version 16.0 (IBM Corp. Released 2016. IBM SPSS Statistics for Windows, Version 24.0. Armonk, NY: IBM Corp) was used for statistical analysis. Quantitative variables were tested for normality using the Shapiro-Wilk test. Descriptive data were presented as the numbers (n), frequencies (%), mean, and standard deviation. The Kruskal-Wallis test was used to determine whether there was a difference between the four groups' distributions of follower numbers and number of posts. If the overall test was determined to be significant, a pairwise comparison using the Wilcoxon signed-rank test was performed. P < 0.05 was considered to reflect statistical significance.

## Results

**Figure 1:**
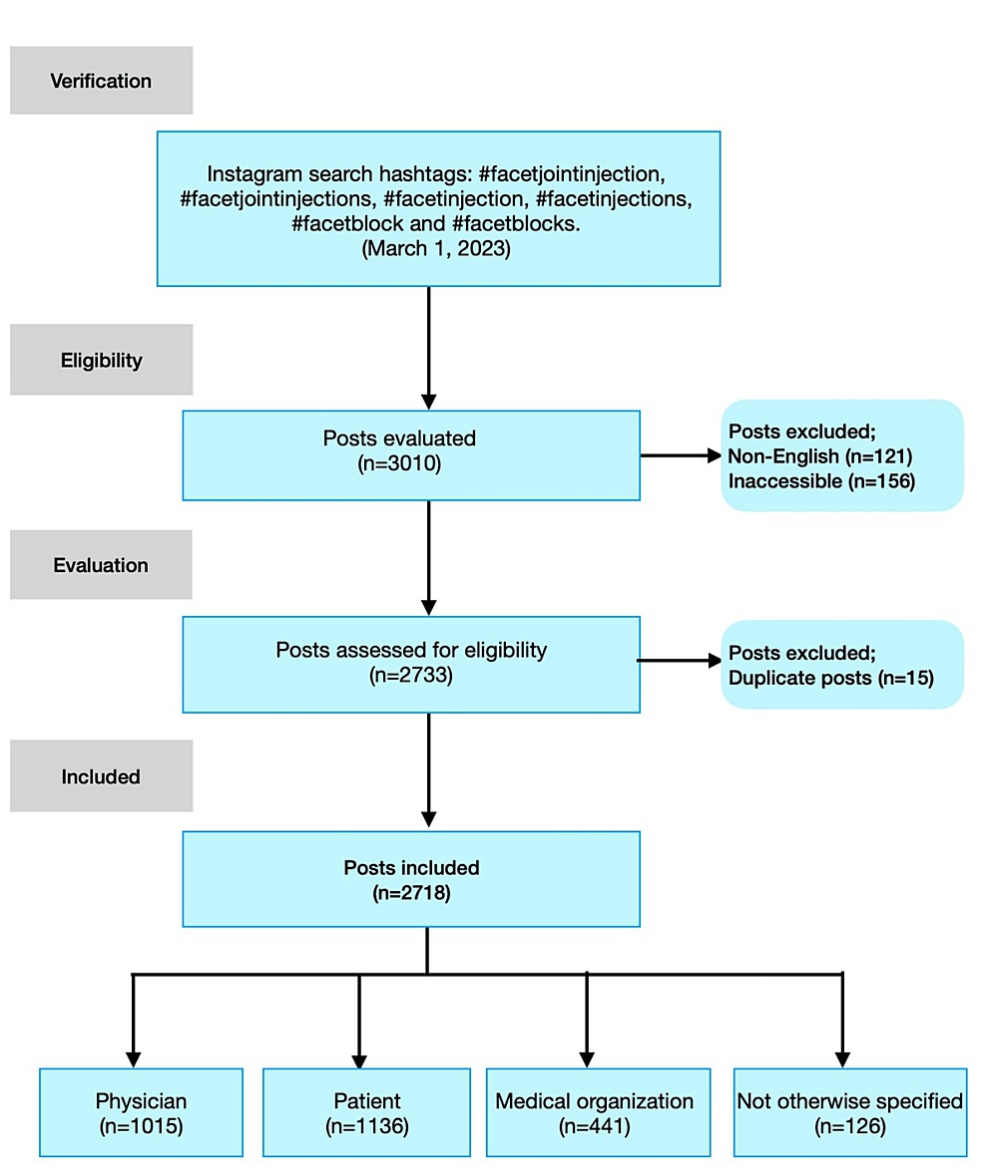
Flow chart of the selection of the posts

A total of 2718 Instagram posts were evaluated. The flowchart for the video selection was demonstrated in Figure 1.

There were 638 different accounts classified as physicians, medical organizations, patients, or not otherwise specified. These accounts were distributed as follows: 43.1% physicians (n=275), 27.11% patients (n=173), 16.3% medical organizations (n=104), and 13.47% not otherwise specified (n=86) (Table 1).

**Table 1:** Instagram post characteristics Values are presented as numbers (%) SD: standard deviation

User	Number of accounts	Number of followers (mean±SD)
Physicians	275 (43.1%)	944,147 (3433.26±12789.44)
Surgeon	189 (68.73%)	752,201 (3979.89±10696.32 )
Non-surgeon	86 (31.27%)	191,946 (2231.93±7554.20 )
Patients	173 (27.11%)	96,838 (559.75±4572.13)
Medical organization	104 (16.3%)	50,110 (481.82±7522.23)
Not otherwise specified	86 (13.47%)	29,071 (338.03±2011.89)

Among the 2718 posts examined, 1136 (41.79 %) were from accounts created by patients, 1015 (37.34%) by physicians, 441 (16.22%) by medical organizations, and 126 (4.63%) were unspecified (Table 2).

**Table 2:** Content analysis of posts SD: standard deviation

User	Total number of posts (mean±SD)	Educational	Experiences
Physician	1015 (3.69±2.16)	72%	28%
Surgeon	731 (3.86±5.35)	75%	25%
Non-surgeon	284(3.30±1.74)	69%	31%
Patient	1136 (6.56±18.47)	2%	98%
Medical organization	441 (4.24±2.76)	92%	8%
Not otherwise specified	126 (1.46±13.15)	-	-

A total of 275 accounts that were connected to patients were experience-based (98%) and educational (2%). The substance of posts by physicians was educational (72%) of the time and personal experiences (28%) of the time. The educational content made up 92% of the hospital-related posts, while the experiential content made up 8% (Table 2).

The study assessed the variation in follower count distribution among four distinct groups, with pairwise comparison indicating a substantial difference in post distribution between patients and physicians (p=0.043), as well as patients and other unspecified groups (p=0.005). Additionally, the study demonstrated a significant distinction in follower distribution between physicians and all other groups examined (Table 3).

**Table 3:** Difference in numbers of posts and numbers of followers *P < 0.05 is considered statistically significant ^a ^Wilcoxon sign rank test was performed for pairwise comparison

	Number of posts (p-value)^a ^	Number of followers (p-value)^a ^
Physician vs patients	0.043*	<0.001*
Physician vs medical organization	0.069	0.006*
Physician vs not otherwise specified	0.060	<0.001*
Patients vs medical organization	0.106	0.924
Patients vs not otherwise specified	0.005*	0.588
Medical organization vs not otherwise specified	0.057	0.852

**Figure 2:**
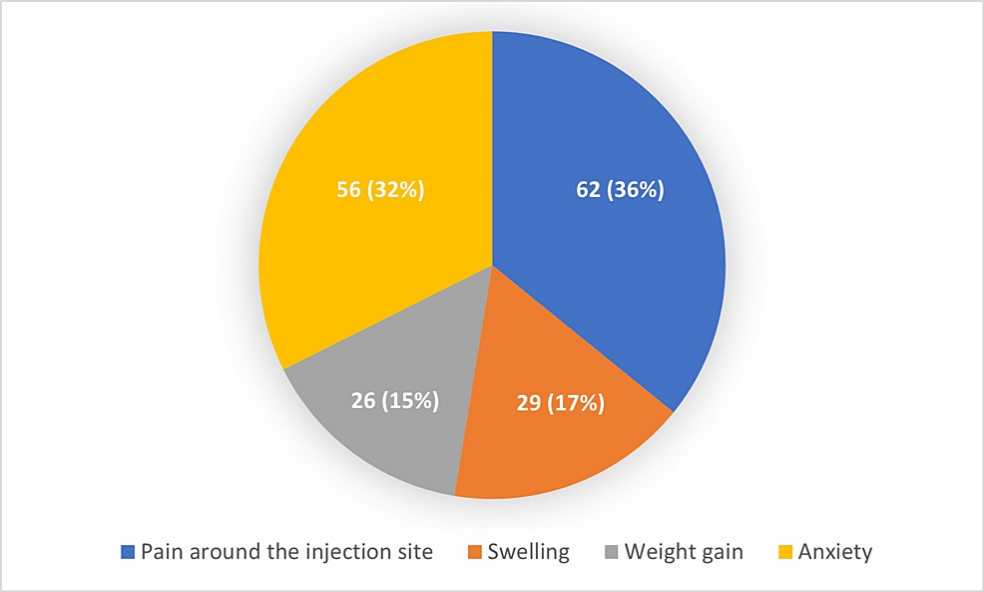
Distribution of reported side effects Data are presented as numbers (%)

The posts were searched for side effects. The side effects expressed by patients were classified by injection pain, swelling, weight gain, and anxiety. A total of 173 accounts mentioned side effects. The reported side effects in the patient's accounts were as follows: pain around the injection site (62, 35.83%), swelling (29, 16.76%), weight gain (26, 15.02%), and anxiety (56, 32.36%) (Figure 2).

## Discussion

In this study, the content relating to the presence of facet joint interventions on the rapidly growing social media platform, Instagram, was assessed. The results of this study demonstrated that physician accounts are the most popular and mostly educational in content. Physicians are widely present on social media; however, when searching for posts about facet joint interventions, the public is more likely to come across a message written by patients. The majority of FJI postings on Instagram were created by patients (41%) and lacked constructs from health belief models. The majority of the posts included in this study were “patient” posts which were real patient experiences or opinions about facet joint interventions. Along with pain around the injection site, anxiety was a major side effect reported by patients.

FJI is a minimal-invasive procedure performed to diagnose and treat pain related to the facet joints in the spine. In order to perform FJI with accuracy and safety, it is important to visualize bony structures. Currently, FJI is carried out under ultrasound guidance and fluoroscopy guidance. Facet joints are small joints located between the vertebrae in the spine that help provide stability and allow for movement. Facet joint block involves injecting a small amount of local anesthetic and/or steroid medication into the affected facet joint. While local anesthetics inhibit nerve conduction and excitation, steroids play a role in inhibiting inflammation. Diagnostic FJI is a reliable method of identifying pain that originates from the facet joint and can potentially provide patients with pain relief and inform future interventions or treatments. In general, FJIs are considered to be procedures with a moderate- to low-risk profile [8,9].

The Internet has revolutionized the world and resulted in innovative advances in the education and dissemination of medical information. It has become one of the most important sources of information for health issues with the capacity to rapidly sort through a huge amount of information [10,11]. Instagram is a widely used visual-based social media networking site, boasting over one billion members worldwide. Users of Instagram have the option to publish both images and videos through a single post, reels, stories, or IGTV. Further, Instagram provides users with the ability to connect with individuals across the world through various features, such as likes, comments, direct messages, and hashtags. Although Instagram was not created for the dissemination of health information or medical education, the Internet revolution has had an impact on health platforms, making it one of the most popular sources of health data.

Similar to previous studies investigating the influence of social media on health conditions and interventions, the majority of posts were created by individual accounts and shared personal experiences [12,13]. This finding shows that patients use the Internet to disseminate medical information. In general, patient experiences may be useful for prospective patients searching for real-life feedback on facet joint procedures. A greater understanding of the experiences of patients undergoing facet joint interventions that are documented on this platform may provide beneficial information for prospective patients seeking real-life opinions about facet joint interventions. However, the viewpoint of patients who have undergone facet joint interventions may be biased, adversely affecting the potential patients and causing the dissemination of unfiltered, frequently incorrect information. The lack of posts authored by medical professionals or organizations indicates that Instagram may represent a missed opportunity for the dissemination of reliable information on facet joint interventions.

This present study showed that 32% of patients who underwent facet joint interventions reported anxiety in their posts. Many patients are not aware of or are not accustomed to interventional pain management, a different type of treatment option. The American Society of Interventional Pain Physicians Guidelines recommend moderate sedation to enhance patient comfort and manage anxiety for therapeutic facet joint interventions [14]. Several studies investigated the impact of psychological variables in facet joint blocks. It has been reported that patients with psychological or emotional factors may not respond well to surgical and interventional techniques and may be difficult to diagnose accurately [15-18]. Manchikanti et al. found that false-positive responses to diagnostic FJIs were more prevalent in the cervical region in patients with major depression [19]. Additionally, Wasan et al. investigated the effect of psychological factors on lumbar and cervical facet joint pain and reported that patients with low psychopathology disorders experienced greater improvements in pain compared to patients with high psychopathology disorders [20]. A growing body of evidence suggests that psychosocial factors can significantly impact the outcome of interventional procedures [14,21,22]. The findings indicate that patients may not be receiving sufficient information regarding facet joint interventions prior to undergoing treatment. Additional education or counseling may reduce the rate of anxiety which was reported at 32% in this present study.

This article has several limitations to be noted. Firstly, the evaluation was completed in a single day. As Instagram is a dynamic platform, there is a possibility that this study may have missed some of the online discussions that have occurred on Instagram. Additionally, different keywords regarding facet joint interventions could be searched. As Instagram does not comprise the demographic characteristics of users such as age range, gender, and other relevant medical history, these variables could not be investigated. Another limitation of this study is that only English posts were included. However, English is a universal language, and English-language information may be obtained from anywhere in the world. Finally, since patients can search for information on platforms other than Instagram, additional research evaluating and comparing various platforms should be conducted.

## Conclusions

This study is the first to investigate facet joint interventions on Instagram. This study's findings highlight Instagram's potential as a platform for raising public awareness of facet joint interventions. The utilization of the Internet for health information is increasing. However, access to health information does not guarantee comprehension due to the variability of the available information. The results of this study indicate that Instagram is not commonly used by medical organizations and physicians as a means of communicating information about FJIs to patients. Furthermore, the results suggest that posts authored by patients are more likely to be encountered by the general public when searching for information on facet joint interventions. Due to a lack of information and their anxiety about the unknown, patients have voiced hesitation about undergoing FJIs. To address this issue, it is the responsibility of physicians to enhance the accessibility of accurate information for patients in order to alleviate their anxiety. Considering that accessibility of Instagram may provide a beneficial platform for educational content on interventional procedures, trustworthy Instagram accounts created by well-known pain medicine societies should be encouraged to produce more online content that clearly states learning objectives on facet joint interventions. Credible posts with accurate information and clear, high-quality images and videos with proper scientific commentary should be uploaded by reputable professional pain medicine societies and qualified specialists to improve the quality of online health information on facet joint interventions. Permitting the sharing of health-related information in digital environments under the supervision of a reputable institution has the potential to yield advantages for public health. Further research is needed to better understand the full potential of Instagram as a health communication tool for facet joint interventions and also for different kinds of medical procedures.
